# [Retracted] Vascular endothelial growth factor participates in modulating the C6 glioma-induced migration of rat bone marrow-derived mesenchymal stem cells and upregulates their vascular cell adhesion molecule-1 expression

**DOI:** 10.3892/etm.2026.13111

**Published:** 2026-02-24

**Authors:** Zhiqiang Gao, Peng Cheng, Yixue Xue, Yunhui Liu

Exp Ther Med 4:993–998, 2012; DOI: 10.3892/etm.2012.707

Following the publication of this paper, it was drawn to the Editor's attention by a concerned reader that, regarding the immunofluorescence analysis data shown in Fig. 5A and C, these data panels were strikingly similar to data that had appeared in Fig. 3 in a previous article featuring some of the same authors (Cheng P, Gao Z-q, Liu Y-h and Xue Y-x: Platelet-derived growth factor BB promotes the migration of bone marrow-derived mesenchymal stem cells towards C6 glioma and up-regulates the expression of intracellular adhesion molecule-1. Neurosci Lett 451: 52-56, 2009). Moreover, the experimental conditions reported in these two papers were different: The above paper was concerned with VCAM-1 expression, whereas the same data were described as being intended to represent ICAM-1 expression in the *Neuroscience Letters* paper.

Owing to the fact that the abovementioned data had been re-used in the above paper in an unrelated experimental context, the Editor of *Experimental and Therapeutic Medicine* has decided that this paper should be retracted from the Journal on account of a lack of confidence in the presented data. The authors were asked for an explanation to account for these concerns, but the Editorial Office did not receive a reply. The Editor apologizes to the readership for any inconvenience caused.

